# Supplementary material for: Sarm1 Deletion, but Not WldS, Confers Lifelong Rescue in a Mouse Model of Severe Axonopathy
Source: Cell Rep. 2017 Oct 3;21(1):10–6. doi: 10.1016/j.celrep.2017.09.027 (PMC5640801; doi:10.1016/j.celrep.2017.09.027)
Supplement: Document S1. Figures S1 and S2 and Table S1 [file mmc1.pdf]

Cell Reports, Volume *21*

Supplemental Information

Sarm1 Deletion, but Not *Wld*^{S}, Confers Lifelong Rescue in a Mouse Model of Severe Axonopathy

Jonathan Gilley, Richard R. Ribchester, and Michael P. Coleman

cross	viable offspring (>P10)		
	Genotype	***Obs.***	*Exp.*
Nmnat2$^{+/gtE}$;Wld$^{S/S}$ x *Nmnat2$^{+/gtE}$;Wld$^{S/S}$*	*Wld$^{S/S}$*	**67**	68.5
	Nmnat2$^{+/gtE}$;Wld$^{S/S}$	**144**	137
	Nmnat2$^{gtE/gtE}$;Wld$^{S/S}$	**63**	68.5

χ^2 test p = 0.660

cross	viable offspring (>P10)		
	Genotype	***Obs.***	*Exp.*
Nmnat2$^{+/gtE}$;Sarm1$^{-/-}$ x *Nmnat2$^{+/gtE}$;Sarm1$^{-/-}$*	*Sarm1$^{-/-}$*	**32**	31.25
	Nmnat2$^{+/gtE}$;Sarm1$^{-/-}$	**61**	62.5
	Nmnat2$^{gtE/gtE}$;Sarm1$^{-/-}$	**32**	31.25

χ^2 test p = 0.965

Table S1. Mendelian ratios of viable offspring from *Nmnat2$^{+/gtE}$;Wld$^{S/S}$* and *Nmnat2$^{+/gtE}$;Sarm1$^{-/-}$* crosses (updated). Related to Figure 1.

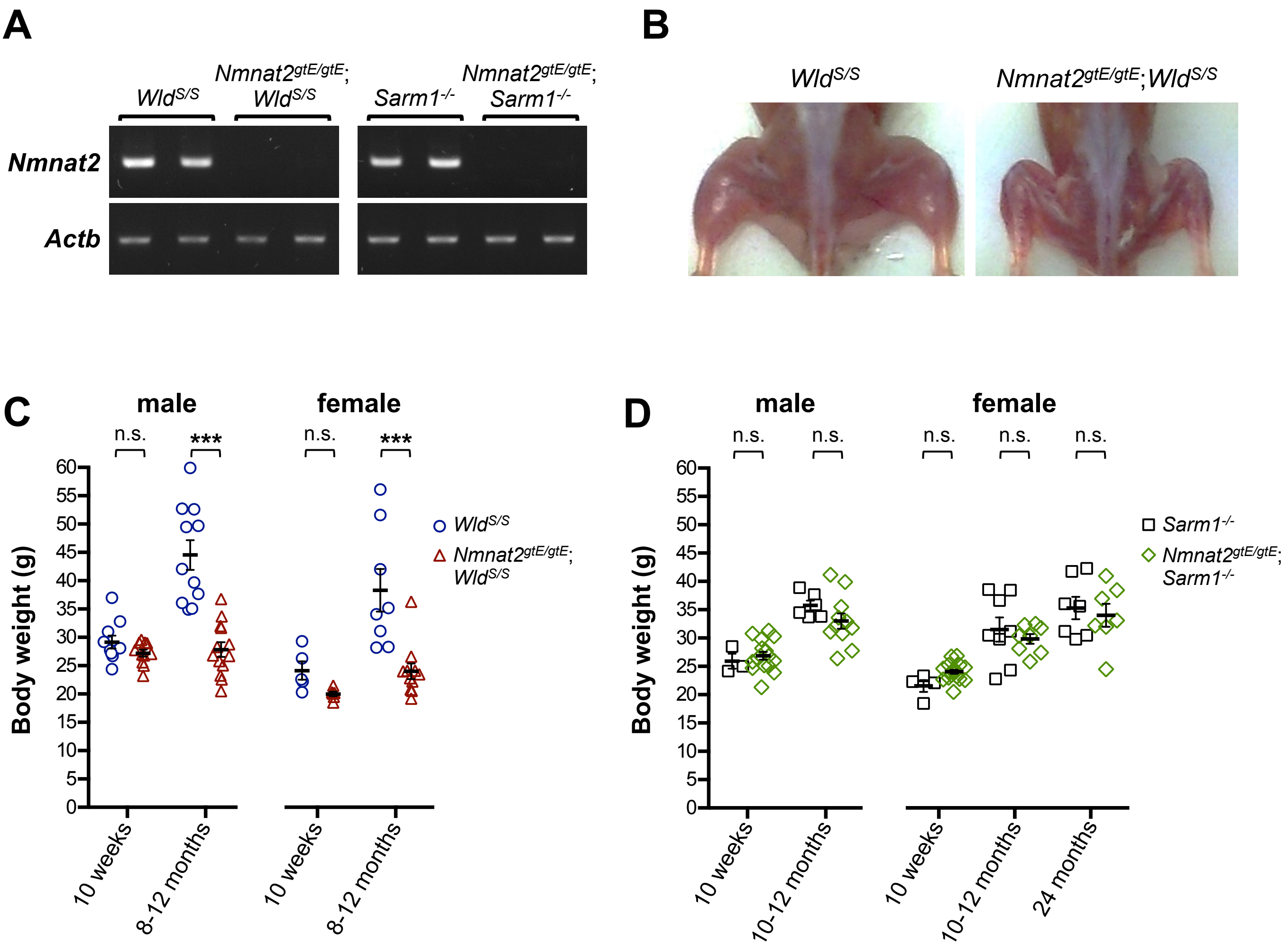

Figure S1. Muscle atrophy and an absence of weight gain in *$Nmnat2^{gtE/gtE}$;$Wld^{S/S}$* mice but not *$Nmnat2^{gtE/gtE}$;$Sarm1^{-/-}$* mice. Related to Figure 1. (**A**) RT-PCR analysis of *Nmnat2* mRNA levels in brains of 10 to 12-month-old mice of the indicated genotypes. *Actb* acts as a sample reference. Images are representative of n = 3 mice per genotype. Cycle numbers were adjusted for *Nmnat2* detection such that band intensity is close to saturation for the *$Wld^{S/S}$* and *$Sarm1^{-/-}$* control samples to emphasize the relative absence of *Nmnat2* mRNA in the *$Nmnat2^{gtE/gtE}$;$Wld^{S/S}$* and *$Nmnat2^{gtE/gtE}$;$Sarm1^{-/-}$* samples. (**B**) Widespread hindlimb muscle wasting in *$Nmnat2^{gtE/gtE}$;$Wld^{S/S}$* mice relative to matched *$Wld^{S/S}$* controls. Representative 6-month-old female mice are shown but the phenotype invariantly develops from 3-5 months onwards in both male and female *$Nmnat2^{gtE/gtE}$;$Wld^{S/S}$* mice. (**C** and **D**) Body weights of male and female *$Wld^{S/S}$* and *$Nmnat2^{gtE/gtE}$;$Wld^{S/S}$* mice (**C**) and *$Sarm1^{-/-}$* and *$Nmnat2^{gtE/gtE}$;$Sarm1^{-/-}$* mice (**D**) at the indicated ages. Individual values with means ±SEM are plotted (n.s. not significant [$p > 0.05$] or ***$p < 0.001$ in two-way ANOVA with Bonferroni's multiple comparisons, separate tests for males and females).

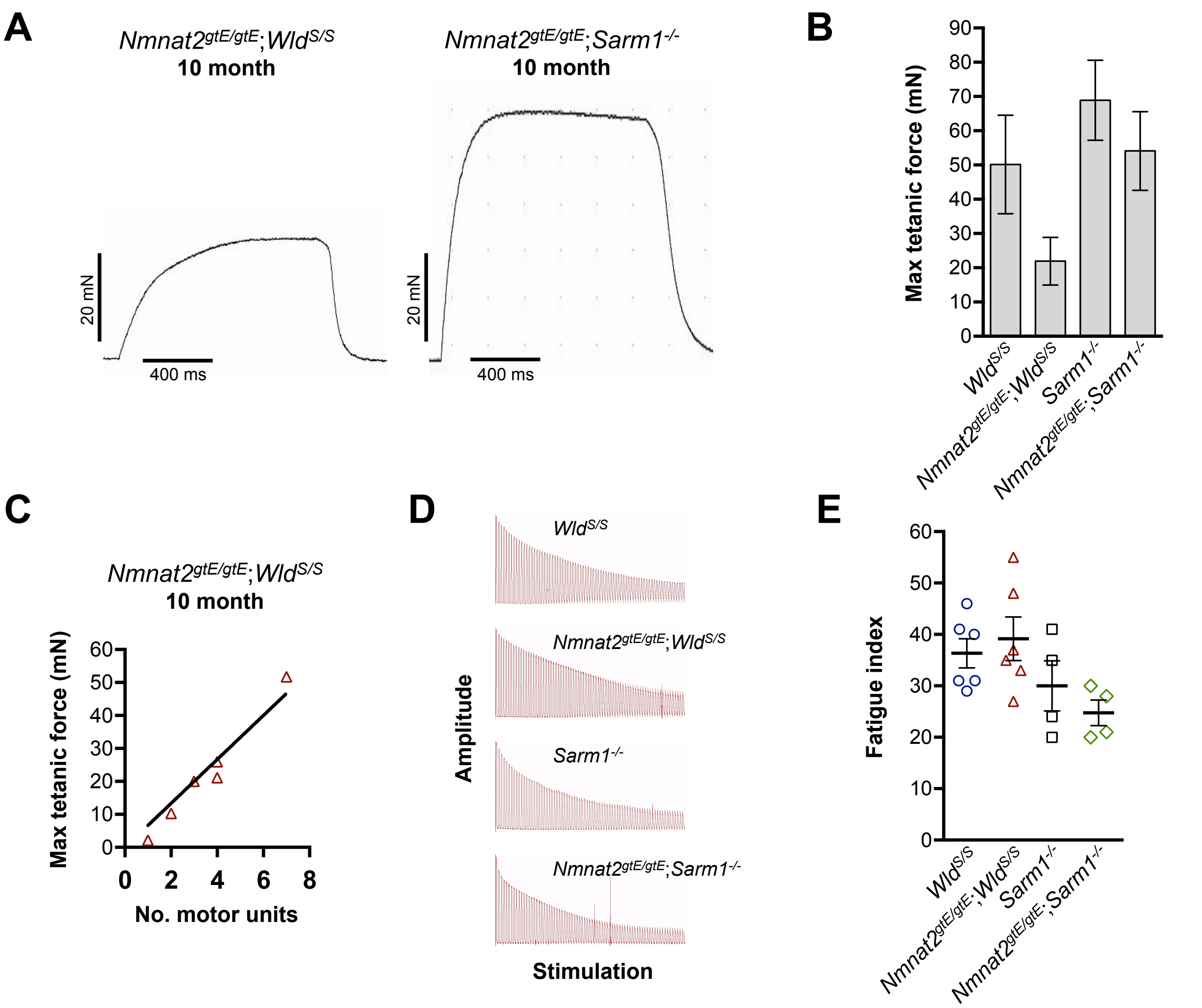

Figure S2. Motor unit numbers are reduced in 10 month *Nmnat2$^{gtE/gtE}$;Wld$^{S/S}$* FDB muscles but remaining units function normally. Related to Figure 2. (**A**) Representative maximum tetanic tension response (50 Hz stimulation for 1 s) of FDB muscle preparations from 10-month-old male *Nmnat2$^{gtE/gtE}$;Wld$^{S/S}$* and *Nmnat2$^{gtE/gtE}$;Sarm1$^{-/-}$* mice. (**B**) Maximum tetanic force of FDB muscles from10-month-old male mice of the genotypes listed. Data are mean ±SEM (n = 6 muscles from three mice, except for n = 4 muscles from two *Sarm1$^{-/-}$* mice). *Nmnat2$^{gtE/gtE}$;Wld$^{S/S}$* force is noticeably reduced compared to the other genotypes due to reduced numbers of motor units (Fig. 3E), although substantial intra-genotype variation means that group sizes are not sufficient for statistical significance (one-way ANOVA, $p > 0.05$). (**C**) Correlation between (reduced) numbers of motor units in *Nmnat2$^{gtE/gtE}$;Wld$^{S/S}$* FDB muscle explants and maximum tetanic force. Spearman correlation $r = 0.986$ ($p = 0.0056$). This suggests that, although different numbers of motor units remain in individual 10-month-old *Nmnat2$^{gtE/gtE}$;Wld$^{S/S}$* FDB muscles, those that are present are similar in size. (**D**) Representative fatigue profiles of FDB muscle explants of the four genotypes studied (as indicated) elicited by 50 Hz tetanic stimulation for 3 s, at intervals of 5s (gaps between each tetanus were clipped). (**E**) Fatigue resistance of FDB muscle explants for 10-month-old mice of the genotypes listed. Individual values with means ±SEM are plotted (same groups sizes as in B). The index of fatigue resistance is the stimulus number that produced a tetanic force equal to 50% of the initial force. Fatigueability of *Nmnat2$^{gtE/gtE}$;Wld$^{S/S}$* and *Nmnat2$^{gtE/gtE}$;Sarm1$^{-/-}$* FDB muscles is not significantly different from matched *Wld$^{S/S}$* or *Sarm1$^{-/-}$* controls (one-way ANOVA, $p > 0.05$). However, grouping muscles based on *Wld$^{S/S}$* or *Sarm1$^{-/-}$* status (i.e. excluding *Nmnat2* status) suggests genetic background does influence fatigueability (*t* test for *Wld$^{S/S}$* and *Nmnat2$^{gtE/gtE}$;Wld$^{S/S}$* versus *Sarm1$^{-/-}$* and *Nmnat2$^{gtE/gtE}$;Sarm1$^{-/-}$*, $p = 0.0126$).
