# Supplementary figures and images for: Sarm1 Deletion, but Not WldS, Confers Lifelong Rescue in a Mouse Model of Severe Axonopathy
Source: Cell Rep. 2017 Oct 3;21(1):10–6. doi: 10.1016/j.celrep.2017.09.027 (PMC5640801; doi:10.1016/j.celrep.2017.09.027)

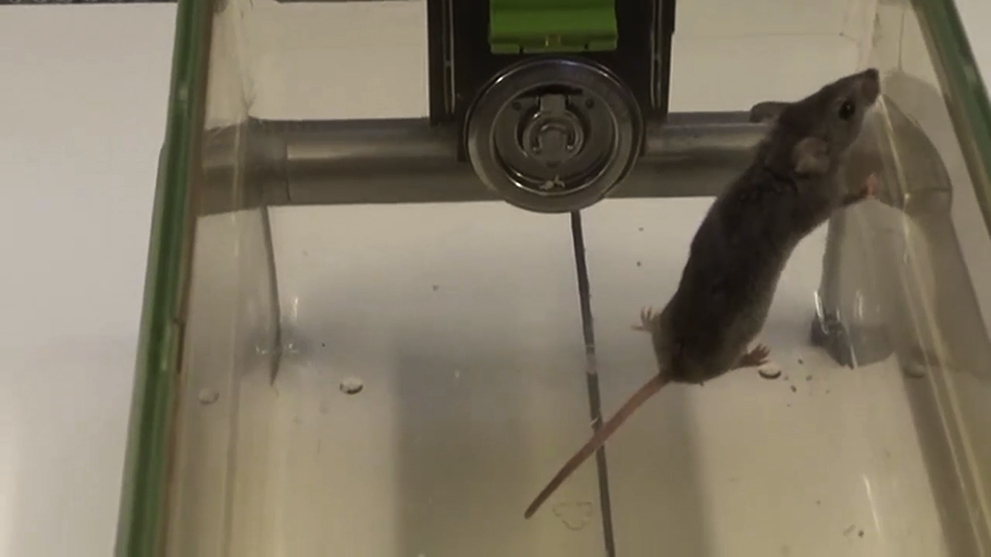

Supplement: Movie S1. Movie of Representative 8- to 9.5-Month-Old Female Nmnat2gtE/gtE;WldS/S and Mice, Related to Figure 1 — Movie highlighting the impaired hindlimb function in Nmnat2gtE/gtE;WldS/S mice but not Nmnat2gtE/gtE;Sarm1−/− mice or controls. [file mmc2.jpg]

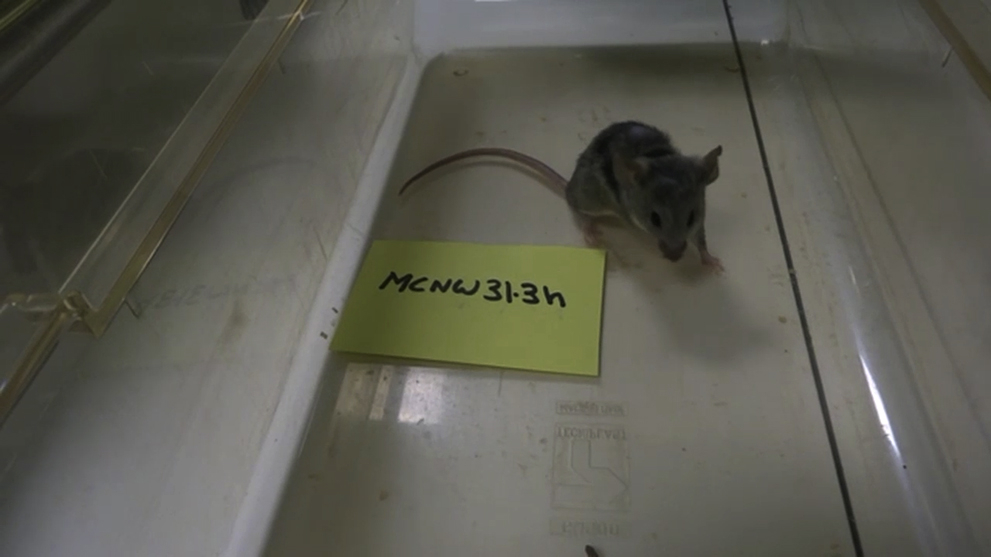

Supplement: Movie S2. Movie of Representative 8- to 9.5-Month-Old Female Nmnat2gtE/gtE;WldS/S and Mice, Related to Figure 1 — Movie highlighting the impaired hindlimb function in Nmnat2gtE/gtE;WldS/S mice but not Nmnat2gtE/gtE;Sarm1−/− mice or controls. [file mmc3.jpg]

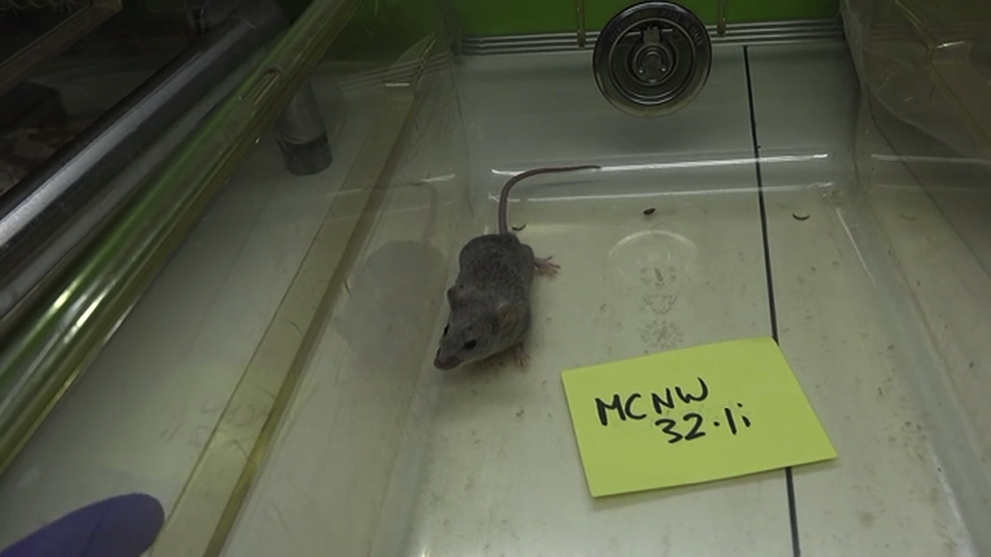

Supplement: Movie S3. Movie of Representative 8- to 9.5-Month-Old Female Nmnat2gtE/gtE;WldS/S and Mice, Related to Figure 1 — Movie highlighting the impaired hindlimb function in Nmnat2gtE/gtE;WldS/S mice but not Nmnat2gtE/gtE;Sarm1−/− mice or controls. [file mmc4.jpg]

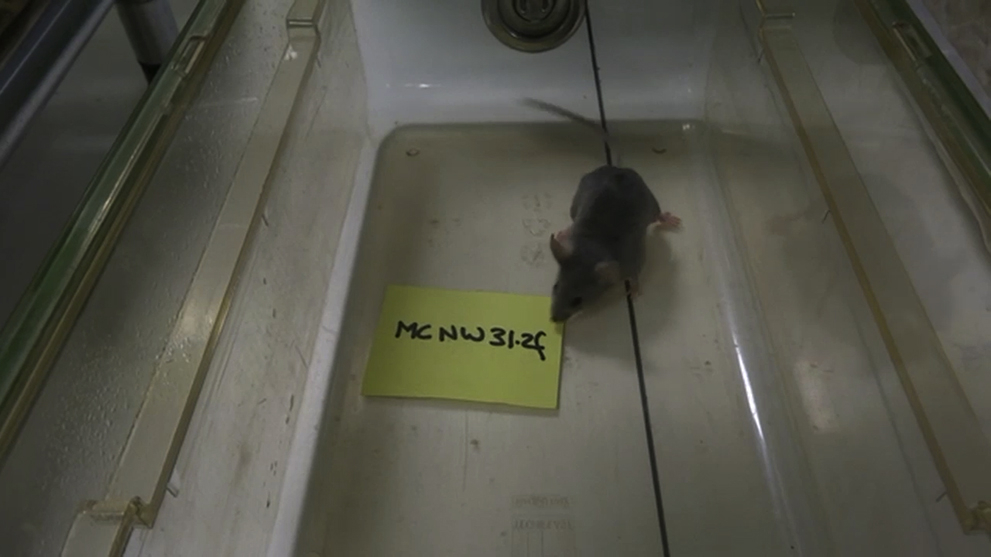

Supplement: Movie S4. Movie of Representative 8- to 9.5-Month-Old Female WldS/S and Mice, Related to Figure 1 — Movie highlighting the impaired hindlimb function in Nmnat2gtE/gtE;WldS/S mice but not Nmnat2gtE/gtE;Sarm1−/− mice or controls. [file mmc5.jpg]

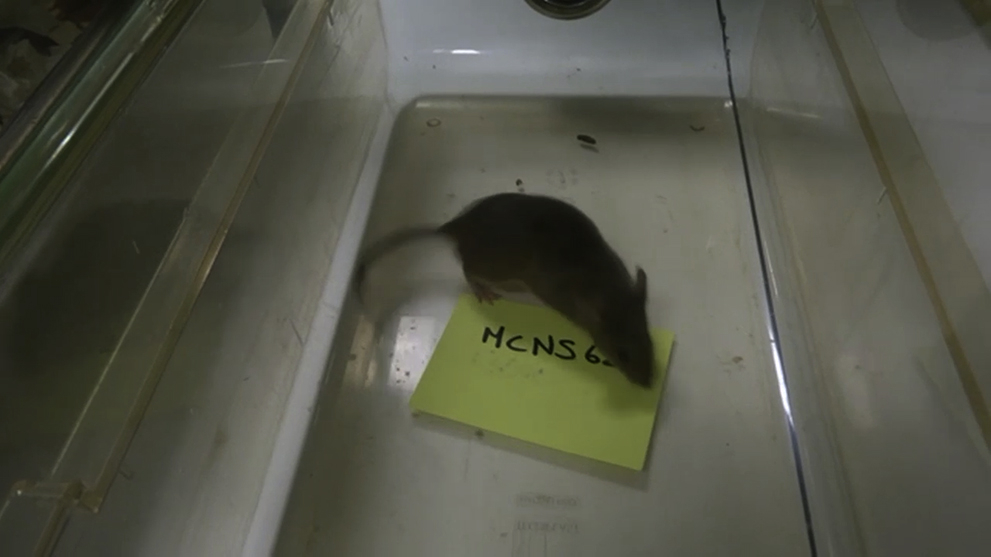

Supplement: Movie S5. Movie of Representative 8- to 9.5-Month-Old Female Nmnat2gtE/gtE;Sarm1−/− and Mice, Related to Figure 1 — Movie highlighting the impaired hindlimb function in Nmnat2gtE/gtE;WldS/S mice but not Nmnat2gtE/gtE;Sarm1−/− mice or controls. [file mmc6.jpg]

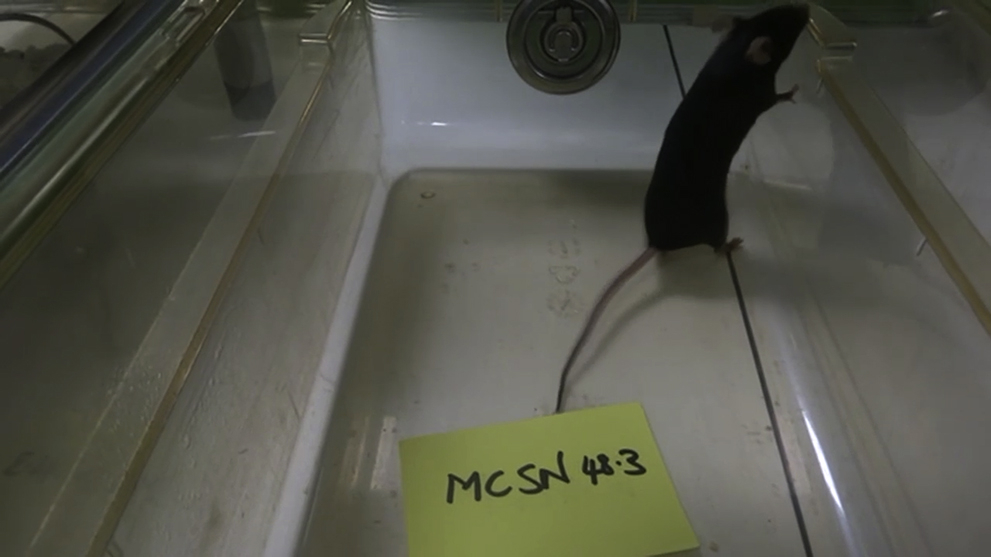

Supplement: Movie S6. Movie of Representative 8- to 9.5-Month-Old Female Sarm1−/− and Mice, Related to Figure 1 — Movie highlighting the impaired hindlimb function in Nmnat2gtE/gtE;WldS/S mice but not Nmnat2gtE/gtE;Sarm1−/− mice or controls. [file mmc7.jpg]
