# Supplementary material for: Sarm1 Deletion, but Not WldS, Confers Lifelong Rescue in a Mouse Model of Severe Axonopathy
Source: Cell Rep. 2017 Oct 3;21(1):10–6. doi: 10.1016/j.celrep.2017.09.027 (PMC5640801; doi:10.1016/j.celrep.2017.09.027)
Supplement: Document S2. Article plus Supplemental Information [file mmc8.pdf]

Cell Reports

Report

Sarm1 Deletion, but Not *Wld*^{S}, Confers Lifelong Rescue in a Mouse Model of Severe Axonopathy

Graphical Abstract

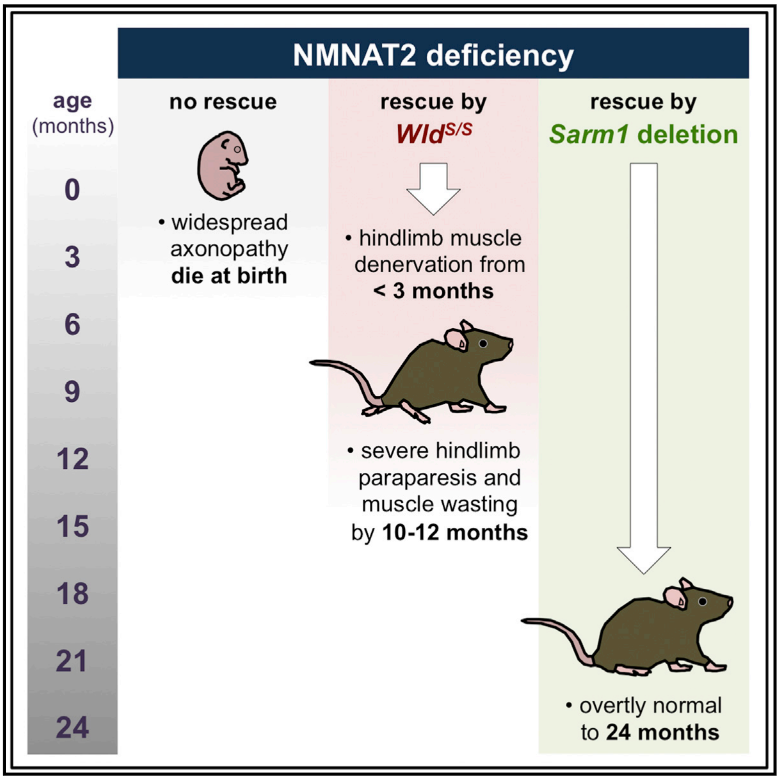

Highlights

- Rescue of an axonopathy model by *Sarm1* deletion or *Wld*^{S} compared in an aging study
- Young adult NMNAT2-deficient mice rescued by *Wld*^{S} develop a hindlimb motor defect
- NMNAT2-deficient mice rescued by *Sarm1* deletion are overtly normal up to 24 months
- SARM1 depletion/inhibition may have analytical and therapeutic advantages over WLD^S

Authors

Jonathan Gilley, Richard R. Ribchester, Michael P. Coleman

Correspondence

jg792@cam.ac.uk

In Brief

Both *Sarm1* deletion and *Wld*^{S} prevent axonopathy and perinatal lethality in NMNAT2-deficient mice. Gilley et al. report that those rescued by *Wld*^{S} develop hindlimb motor problems as young adults, whereas *Sarm1* deletion allows survival to 24 months with no overt defect. These findings have important analytical and therapeutic implications.

Gilley et al., 2017, Cell Reports *21*, 10–16
October 3, 2017 © 2017 The Author(s).
https://doi.org/10.1016/j.celrep.2017.09.027

OPEN ACCESS
CellPress

Cell Reports
Report

Sarm1 Deletion, but Not *Wld*^{S}, Confers Lifelong Rescue in a Mouse Model of Severe Axonopathy

Jonathan Gilley,[1,2,4,*] Richard R. Ribchester,[3] and Michael P. Coleman[1,2]
[1]John van Geest Centre for Brain Repair, Department of Clinical Neurosciences, University of Cambridge, ED Adrian Building, Forvie Site, Robinson Way, Cambridge CB2 0PY, UK
[2]Signalling Programme, Babraham Institute, Babraham Research Campus, Cambridge CB22 3AT, UK
[3]Biomedical Sciences, Euan MacDonald Centre for MND Research and Centre for Discovery Brain Sciences, The University of Edinburgh, 1 George Square, Edinburgh EH8 9JZ, UK
[4]Lead Contact
*Correspondence: jg792@cam.ac.uk
https://doi.org/10.1016/j.celrep.2017.09.027

SUMMARY

Studies with the *Wld*^{S} mutant mouse have shown that axon and synapse pathology in several models of neurodegenerative diseases are mechanistically related to injury-induced axon degeneration (Wallerian degeneration). Crucially, an absence of SARM1 delays Wallerian degeneration as robustly as *Wld*^{S}, but their relative capacities to confer long-term protection against related, non-injury axonopathy and/or synaptopathy have not been directly compared. While *Sarm1* deletion or *Wld*^{S} can rescue perinatal lethality and widespread Wallerian-like axonopathy in young NMNAT2-deficient mice, we report that an absence of SARM1 enables these mice to survive into old age with no overt phenotype, whereas those rescued by *Wld*^{S} invariantly develop a progressive neuromuscular defect in their hindlimbs from around 3 months of age. We therefore propose *Sarm1* deletion as a more reliable tool than *Wld*^{S} for investigating Wallerian-like mechanisms in disease models and suggest that SARM1 blockade may have greater therapeutic potential than WLD^S-related strategies.

INTRODUCTION

Wld^S, a spontaneous mutant mouse allele encoding a fusion protein (WLD^S) with nicotinamide mononucleotide adenylyltransferase (NMNAT) activity, robustly delays injury-induced axon and synapse degeneration (Wallerian degeneration) by locally substituting for loss of the endogenous NMNAT2 isoform (Mack et al., 2001; Gilley and Coleman, 2010; Cohen et al., 2012; Conforti et al., 2014). *Wld*^{S} has been the tool of choice for investigating the molecular basis of axon pathology in animal models of neurodegenerative diseases and has revealed an involvement of Wallerian-like mechanisms in several cases (Conforti et al., 2014). Key steps in this process are thus potential targets for intervention in patients.

Sterile alpha and TIR motif-containing protein 1 (SARM1) acts downstream of NMNAT2 loss to promote axon degeneration (Osterloh et al., 2012; Gerdts et al., 2013; Gilley et al., 2015; Loreto et al., 2015; Walker et al., 2017). Depletion of SARM1 is, to date, the only other manipulation that can delay Wallerian degeneration and related axon degeneration in mice as robustly as exogenous expression of WLD^S or other NMNAT variants (Conforti et al., 2014), but its effectiveness in maintaining the long-term health of axons and synapses in mouse models of axonopathy and/or synaptopathy has not yet been directly compared to *Wld*^{S}. Such a comparison is needed to ascertain the relative usefulness of *Sarm1* deletion in determining whether Wallerian-like mechanisms are involved in models of neurodegeneration and should be informative in terms of therapeutic strategies for those disorders.

An absence of NMNAT2 in mice causes widespread axon truncation during embryogenesis and perinatal lethality (Gilley et al., 2013). Early rescue by *Wld*^{S} (dose dependently) or by *Sarm1* deletion has shown that outgrowth of NMNAT2-deficient axons stalls due to a Wallerian-like degenerative mechanism (Gilley et al., 2013, 2015). Reduced NMNAT2 levels have already been linked to tauopathy in mice and to decreased cognitive function in humans (Ljungberg et al., 2012; Ali et al., 2016), but the severity of the phenotype in mice lacking NMNAT2 suggests a complete lack of the protein is unlikely to directly model any neurodegenerative conditions. Nevertheless, these mice represent a well-defined and robust system for comparing the longer-term protective effects of *Wld*^{S} and *Sarm1* deletion against a severe Wallerian-like axonopathy. While the survival of NMNAT2-deficient mice homozygous for either *Wld*^{S} or a *Sarm1* knockout allele up to 3 months of age with no overt problems initially suggested similarly robust rescue in each case (Gilley et al., 2013, 2015), we now report striking age-dependent differences between the two lines, which are likely to have important experimental and therapeutic implications.

RESULTS

Locomotor Defects and Muscle Atrophy in *Nmnat2*$^{gtE/gtE}$;*Wld*$^{S/S}$ Mice, but Not *Nmnat2*$^{gtE/gtE}$;*Sarm1*$^{-/-}$ Mice

Mice homozygous for the *Nmnat2*^{gtE} gene trap allele, lacking NMNAT2, that are additionally homozygous for *Wld*^{S} or a *Sarm1* knockout allele (*Nmnat2*$^{gtE/gtE}$;*Wld*$^{S/S}$ or *Nmnat2*$^{gtE/gtE}$;*Sarm1*$^{-/-}$ mice, respectively) are born at the expected frequencies and are outwardly indistinguishable from NMNAT2-expressing littermates up to 3 months of age (Table S1) (Gilley et al.,

This is an open access article under the CC BY license (http://creativecommons.org/licenses/by/4.0/).
CrossMark

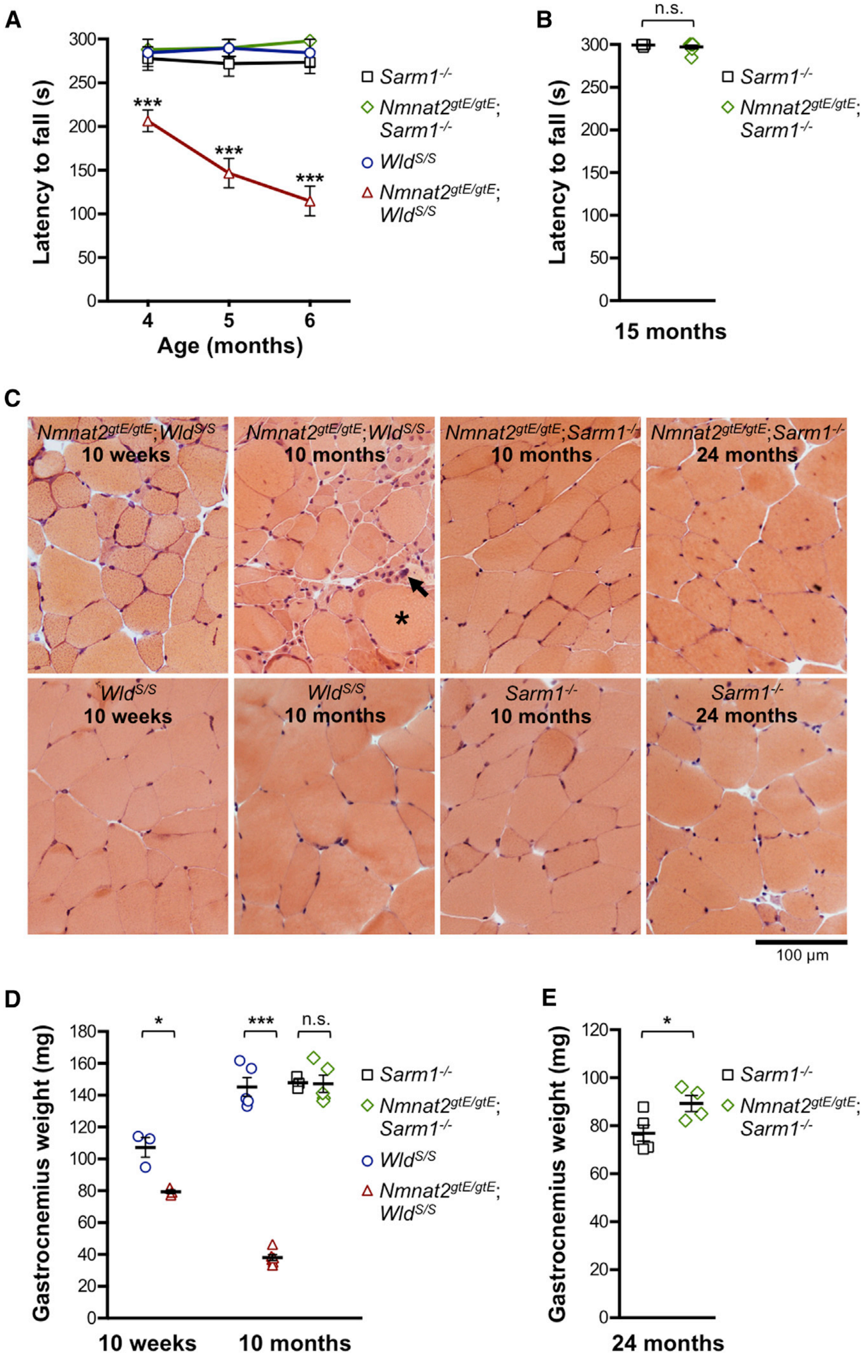

Figure 1. Progressive Locomotor Dysfunction and Hindlimb Muscle Atrophy in ***Nmnat2*$^{gtE/gtE}$;*Wld*$^{S/S}$ Mice, but Not *Nmnat2*$^{gtE/gtE}$;*Sarm1*$^{-/-}$ Mice**

(A and B) Latency to fall in an accelerating Rotarod task for mice of the indicated genotypes and ages. Means ± SEM are plotted (maximum test duration, 300 s) for n = 7/8 male mice of each genotype (A) (***p < 0.001 in two-way ANOVA with Dunnett's multiple comparisons) and n = 7 female mice of each genotype (B) (NS, not significant [p = 0.29] in t test).

(C) Representative transverse sections of gastrocnemius muscle for mice of the selected genotypes and ages (as indicated) stained with H&E. Modest fiber atrophy is seen in *Nmnat2*$^{gtE/gtE}$;*Wld*$^{S/S}$ gastrocnemius at 10 weeks, and more severe atrophy, with centrally located nuclei, hypertrophic fibers (*), and pyknotic nuclear clumps (arrow), is evident at 10 months. Images are representative of male and female mice at 10 weeks and 8–12 months but just female mice at 24 months.

(D and E) Gastrocnemius muscle weights for mice of the indicated genotypes and ages. Individual animal values with means ± SEM are plotted for n = 3–5 male mice per group (D) (*p < 0.05 and ***p < 0.001 in one-way ANOVA with Tukey's multiple comparisons; NS, not significant) and n = 4–5 female mice per group (E) (*p < 0.05 in t test).

See also Movies S1, S2, S3, S4, S5, and S6, Table S1, and Figure S1.

2013, 2015). However, despite continued silencing of the trapped *Nmnat2* alleles in each case (Figure S1A), further aging has revealed clear differences between the lines: *Nmnat2*$^{gtE/gtE}$;*Sarm1*$^{-/-}$ mice remarkably survived for up to 2 years with no noticeable behavioral deficiency or phenotype, whereas *Nmnat2*$^{gtE/gtE}$;*Wld*$^{S/S}$ mice invariantly developed a conspicuous, progressive hindlimb defect from around 3–5 months of age.

The defect in *Nmnat2*$^{gtE/gtE}$;*Wld*$^{S/S}$ mice (male and female) first presented as a modest hindlimb gait abnormality during spontaneous locomotion, but this progressively deteriorated, resulting in mice invariantly dragging their hindlimbs regularly during locomotion from around 6 months onward as a result of worsening paraparesis (Movies S1, S2, S3, and S4). Consistent with this, locomotor ability of *Nmnat2*$^{gtE/gtE}$;*Wld*$^{S/S}$ mice in an accelerating Rotarod task deteriorated rapidly between 4 and 6 months (Figure 1A). Movement became so limited by 10–12 months that it impaired free access to food and water, so *Nmnat2*$^{gtE/gtE}$;*Wld*$^{S/S}$ mice were not aged further. In contrast, locomotor performance of *Nmnat2*$^{gtE/gtE}$;*Sarm1*$^{-/-}$ mice did not decline during the same period (Figure 1A; Movies S5 and S6), and *Nmnat2*$^{gtE/gtE}$;*Sarm1*$^{-/-}$ mice still performed as well as *Sarm1*$^{-/-}$ controls up to at least 15 months (Figure 1B).

Deteriorating locomotor function in *Nmnat2*$^{gtE/gtE}$;*Wld*$^{S/S}$ mice coincided with progressive and widespread wasting of hindlimb muscles (Figure S1B). A specific analysis of gastrocnemius muscle revealed evidence of some muscle fiber atrophy and slightly reduced muscle weight even at 10 weeks in *Nmnat2*$^{gtE/gtE}$;*Wld*$^{S/S}$ mice, before the onset of overt locomotor dysfunction, but this had progressed to severe muscle fiber atrophy and

OPEN ACCESS
CellPress

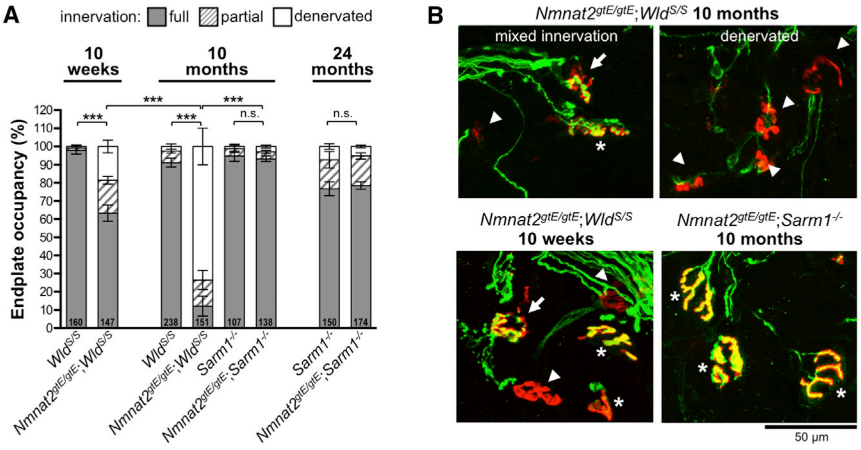

Figure 2. Progressive Loss of NMJ Innervation in Gastrocnemius Muscles of *Nmnat2$^{gtE/gtE}$;Wld$^{S/S}$* Mice, but Not *Nmnat2$^{gtE/gtE}$;Sarm1$^{-/-}$* Mice

(A) Percentage of fully occupied (full), partially occupied (partial), and denervated endplates in gastrocnemius muscles from mice of the indicated genotypes and ages. Endplate occupancy was determined by assessing signal overlap between α-bungarotoxin labeling of acetylcholine receptors in the motor endplate and βIII-tubulin immunostaining of axon terminals (see Experimental Procedures). Total numbers of NMJs analyzed in muscles from 3–5 mice per genotype (male and female) are listed at the base of each column. Means ± SEM of occupancy per animal are plotted (NS, not significant [$p > 0.05$] or ***$p < 0.001$ in one-way ANOVA with Tukey's multiple comparisons of full innervation, selected comparisons).

(B) Representative confocal z series projections of fixed gastrocnemius (Gn) muscle preparations showing merged α-bungarotoxin (red) and βIII-tubulin (green) signals. Endplates in the images are marked as fully occupied (asterisks), partially occupied (arrows), or denervated (arrowheads).

loss of mass by 10 months (Figures 1C and 1D). In contrast, no muscle fiber atrophy or loss of mass was seen in *Nmnat2$^{gtE/gtE}$;Sarm1$^{-/-}$* gastrocnemius up to 24 months (Figures 1C–1E).

Although *Nmnat2$^{gtE/gtE}$;Wld$^{S/S}$* mice did not lose body weight between 10 weeks and 10–12 months, they failed to gain weight as expected (Figure S1C). This presumably reflected loss of muscle mass in the hindlimb being broadly matched by normal weight gain in the upper torso and forelimbs, which appeared largely unaffected. In contrast, developmental weight gain in *Nmnat2$^{gtE/gtE}$;Sarm1$^{-/-}$* mice was comparable to *Sarm1$^{-/-}$* controls up to 24 months (Figure S1D).

Neuromuscular Denervation in *Nmnat2$^{gtE/gtE}$;Wld$^{S/S}$* Mice, but Not *Nmnat2$^{gtE/gtE}$;Sarm1$^{-/-}$* Mice

Changes in neuromuscular junction (NMJ) innervation indicated that the muscle defect in *Nmnat2$^{gtE/gtE}$;Wld$^{S/S}$* mice is neurogenic. Motor endplate occupancy in *Nmnat2$^{gtE/gtE}$;Wld$^{S/S}$* gastrocnemius was found to be moderately reduced at 10 weeks, but consistent with the timing of gastrocnemius muscle fiber atrophy and weight loss in these mice, by 10 months, denervation was extensive, with only around 10% of endplates showing normal innervation (Figures 2A and 2B).

We also investigated motor endplate occupancy in a more distal hindlimb muscle, flexor digitorum brevis (FDB), at 10 months. Although denervation was also evident in this muscle (Figure 3A), it was less severe than in gastrocnemius at the same age. Clear regional variation in the pattern of endplate occupancy was seen in FDB, with discrete zones of normal innervation being found adjacent to zones of complete denervation (Figures 3B and 3C). Isometric tension recordings indicated that this distinctive pattern of NMJ innervation reflects discrete loss of entire motor units (Figures 3D and 3E), with apparently normal function of remaining motor units (Figure S2).

In contrast, and consistent with the lack of locomotor problems, no significant endplate denervation and/or motor unit loss was evident in either gastrocnemius or FDB muscles from 10-month-old *Nmnat2$^{gtE/gtE}$;Sarm1$^{-/-}$* mice (Figures 2, 3A, 3D, 3E, and S2) and innervation remained comparable to *Sarm1$^{-/-}$* controls at 24 months, despite modest age-dependent denervation in both (Figure 2A).

Wld$^{S/S}$ and *Sarm1$^{-/-}$* mice both performed maximally in locomotor tests, and neither showed signs of neuromuscular denervation at the ages studied. Although a direct comparison with wild-type mice will be needed to establish whether more subtle differences in motor function exist in either line, our data suggest that both have broadly normal neuromuscular function. The defect in *Nmnat2$^{gtE/gtE}$;Wld$^{S/S}$* mice thus appears to be specific to a declining inability of WLD^{S} to counter the lack of NMNAT2 in older mice, rather than other intrinsic differences.

No Loss of Myelinated Tibial Nerve Axons in Either *Nmnat2$^{gtE/gtE}$;Wld$^{S/S}$* or *Nmnat2$^{gtE/gtE}$;Sarm1$^{-/-}$* Mice

Despite progressive denervation of motor endplates in hindlimb muscles from *Nmnat2$^{gtE/gtE}$;Wld$^{S/S}$* mice, no concurrent loss of myelinated axons was seen in the tibial nerve (Figure 4A) and axons remained morphologically normal (Figure 4B). A gross assessment revealed that most hindlimb muscles became extensively atrophied in these mice (Figure S1B), so significantly reduced numbers of axons would have been expected, even in a mixed nerve such as this, if motor axon loss was the underlying cause. The age-dependent neuromuscular denervation in *Nmnat2$^{gtE/gtE}$;Wld$^{S/S}$* muscles thus appears to result from selective loss of the distal ends of motor axons and/or their terminals. This mirrors the age-dependent loss of protection of synapses at NMJs after axotomy in homozygous *Wld^{S}* mice, despite continued protection of the main body of the transected axon (Gillingwater et al., 2002).

Counts of myelinated axons in *Nmnat2$^{gtE/gtE}$;Sarm1$^{-/-}$* tibial nerves remained comparable to those of *Sarm1$^{-/-}$* controls, even up to 24 months (Figure 4A), with no age-related axon loss in either group up to this age. We also found no significant axon loss in a separate cohort of wild-type mice (on a related background) up to 24 months (1,514 ± 22 myelinated axons at 1.5 months, compared to 1,473 ± 40 at 24 months). This contrasts a previous study that reported significant loss of myelinated tibial

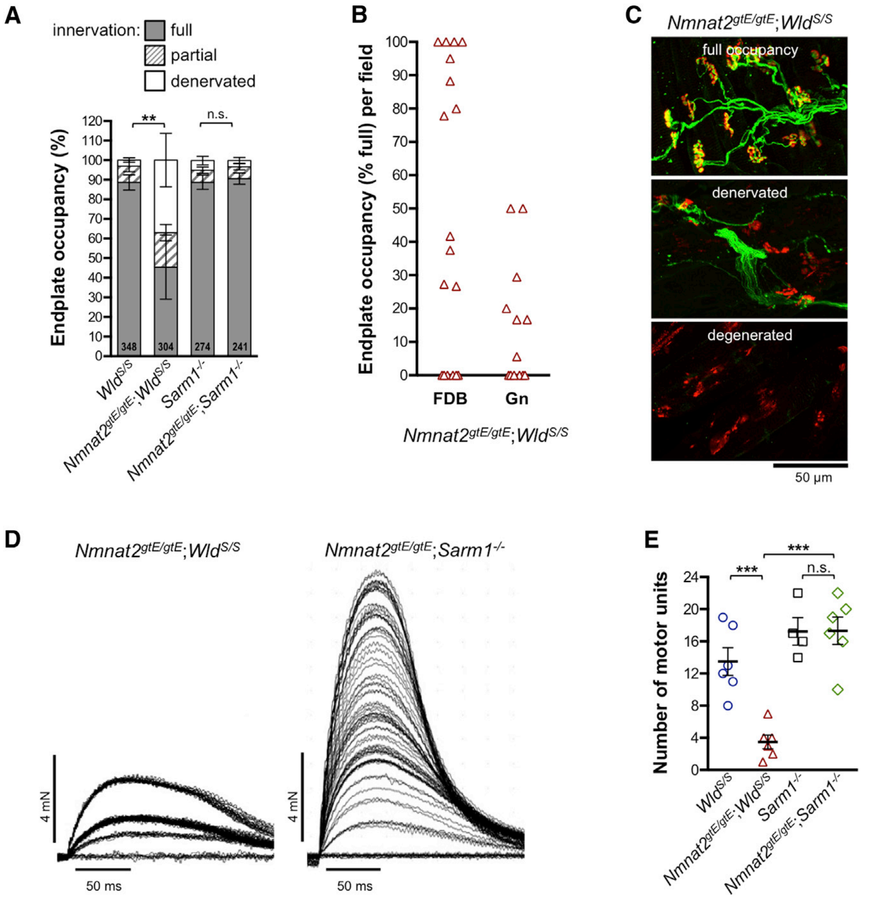

Figure 3. Localized Loss of NMJ Innervation from Distinct Motor Unit Groups in FDB Muscle of *Nmnat2*$^{gtE/gtE}$;*Wld*$^{S/S}$ Mice

(A) Percentage of fully occupied (full), partially occupied (partial), and denervated endplates in FDB muscles of 10-month-old mice of the indicated genotypes (method as in Figure 2A). Total numbers of NMJs analyzed in muscles from 5–9 mice per genotype (male and female) are listed at the base of each column. Means ± SEM of occupancy per animal are plotted (NS, not significant [$p > 0.05$] or **$p < 0.01$ in one-way ANOVA with Tukey's multiple comparisons of full innervation, selected comparisons).

(B) Percentage of full occupancy of endplates per imaged field revealed a zonal pattern of denervation in *Nmnat2*$^{gtE/gtE}$;*Wld*$^{S/S}$ FDB at 10 months, with more widespread denervation in gastrocnemius (Gn). Fields with normal levels of full occupancy (80%–100%) were relatively common in FDB (7/18) but absent in gastrocnemius (average of 16.9 and 11.6 endplates per field, respectively).

(C) Representative confocal z series projections of fixed FDB muscle preparations showing merged α-bungarotoxin (red) and βIII-tubulin (green) signals, highlighting regional variability in *Nmnat2*$^{gtE/gtE}$;*Wld*$^{S/S}$ endplate occupancy at 10 months. In addition to regions with widespread full occupancy (top panel) or denervation (middle panel), there were areas containing degenerated endplates (bottom panel, not included in quantifications).

(D) Isometric twitch tension recordings for representative *Nmnat2*$^{gtE/gtE}$;*Wld*$^{S/S}$ and *Nmnat2*$^{gtE/gtE}$;*Sarm1*$^{-/-}$ FDB muscle-tibial nerve preparations showing progressive, discrete recruitment of motor units (incremental steps in twitch tension) during continuously graded stimulation of the nerve.

(E) Motor unit numbers in FDB muscles from 10-month-old male mice of the indicated genotypes (n = 4 or 6 muscles from 2 or 3 mice as shown). Individual values and means ± SEM are plotted (NS, not significant [$p > 0.99$] or ***$p < 0.001$ in one-way ANOVA with Tukey's multiple comparisons, selected comparisons). See also Figure S2.

nerve axons by 24 months in wild-type mice (Valdez et al., 2010), although this could simply reflect strain differences.

DISCUSSION

To date, *Wld*^{S} has been the preferred tool for assessing the involvement of Wallerian-like axon and synapse degeneration in rodent models of neurodegeneration (Conforti et al., 2014). However, the relatively short-term preservation of neuromuscular innervation by *Wld*^{S} in hindlimb muscles of *Nmnat2*$^{gtE/gtE}$ mice raises the possibility that this strategy might have greatly underestimated the involvement of Wallerian-like mechanisms in some models. Likely candidates are wabbler-lethal (*Atp8a2*$^{wl/wl}$) and gracile axonal dystrophy (*Uchl1*$^{gad/gad}$) mice, in which *Wld*^{S} robustly protects (proximal) axons but does not rescue neuromuscular dysfunction (Mi et al., 2005; Zhu et al., 2012). Human SOD1^{G37R}, SOD1^{G85R}, and SOD1^{G93A} transgenic mouse models of amyotrophic lateral sclerosis (ALS) are also candidates, although *Wld*^{S} largely fails to protect axons in these models, suggesting that unrelated degenerative mechanisms contribute substantially to disease signs (Vande Velde et al., 2004; Fischer et al., 2005). Because SARM1 deficiency confers longer-lasting preservation of NMJ innervation in NMNAT2-deficient mice than *Wld*^{S}, it could confer a better outcome in these or related models.

We consider that prolonged preservation of *Nmnat2*$^{gtE/gtE}$;*Sarm1*$^{-/-}$ motor axon terminals, compared to those in *Nmnat2*$^{gtE/gtE}$;*Wld*$^{S/S}$ mice, might reflect that local availability of WLD^S, which is required for protection (Beirowski et al., 2009; Cohen et al., 2012), is likely to be subject to a variety of influences, whereas protection conferred by an absence of SARM1 will be invariant. Global expression of WLD^S in homozygous *Wld*^{S} mice does not diminish significantly with age up to 12 months (Gillingwater et al., 2002), but we propose that normal changes in physiology, from as young as 2 months of age, could alter the stability, delivery, or activity of WLD^S in *Nmnat2*$^{gtE/gtE}$;*Wld*$^{S/S}$ motor axon terminals, or otherwise alter the local environment, such that it can no longer effectively substitute for the lack of NMNAT2 to promote survival. More widespread NMJ denervation in gastrocnemius compared to FDB in *Nmnat2*$^{gtE/gtE}$;*Wld*$^{S/S}$ mice suggests that changes specific to different muscle or motor unit types are more critical to the loss of

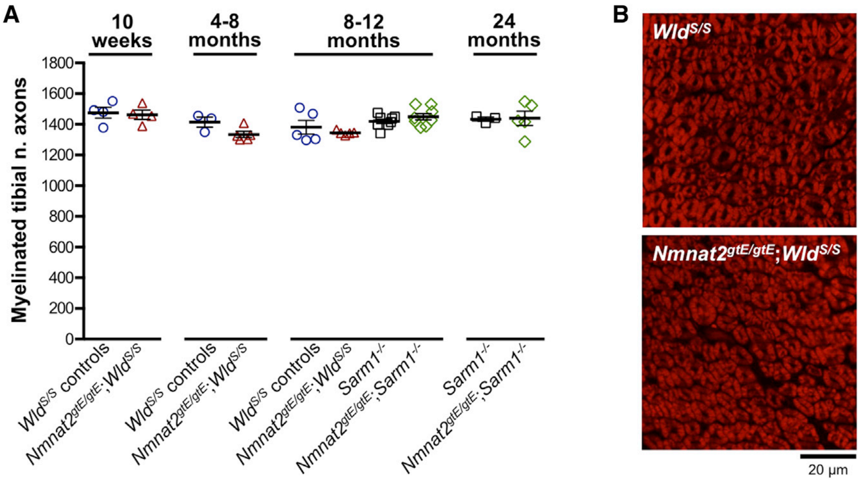

Figure 4. No Loss of Myelinated Tibial Nerve Axons in Either *Nmnat2*$^{gtE/gtE}$;*Wld*$^{S/S}$ or *Nmnat2*$^{gtE/gtE}$;*Sarm1*$^{-/-}$ Mice

(A) Numbers of myelinated axons in tibial nerve (mid-calf level) from mice of the indicated genotypes and ages (*Wld*$^{S/S}$ control groups include some *Nmnat2*$^{+/gtE}$;*Wld*$^{S/S}$ mice that were indistinguishable from *Wld*$^{S/S}$ mice). Individual values (n = 3–8, as shown, male and female) and means ± SEM are plotted. No statistically significant differences were identified between groups (one-way ANOVA with Tukey's multiple comparisons).

(B) FluoroMyelin red-stained tibial nerve cross-sections from 10-month-old *Wld*$^{S/S}$ and *Nmnat2*$^{gtE/gtE}$;*Wld*$^{S/S}$ mice (representative of n = 5 each genotype). No structural differences are evident, even though *Nmnat2*$^{gtE/gtE}$;*Wld*$^{S/S}$ mice have an advanced neuromuscular defect at this age.

WLD^{S}-mediated protection than those relating to axon length (FDB being more distal). These considerations will similarly apply to the age-dependent loss of protection of motor axon terminals after axotomy (and the resulting NMNAT2 loss) in homozygous *Wld*^{S} mice (Gillingwater et al., 2002). Annulospiral (sensory) nerve endings in the muscle remained protected in older mice in that context, suggesting a motor-specific defect (Oyebode et al., 2012). Although we have seen qualitative preservation of annulospiral endings in *Nmnat2*$^{gtE/gtE}$;*Wld*$^{S/S}$ FDB at 10 months (not shown), a comprehensive analysis of sensory innervation will be required to determine whether sensory endings in general are better preserved than motor axon terminals.

A model in which sustaining the effective potency of WLD^{S} locally is required for its protective effects leaves open the possibility that *Sarm1* deletion may be more effective than *Wld*^{S} at rescuing symptoms in models of other types of neurodegenerative disease, not just those with early neuromuscular symptoms. If the disease-causing defect in a given model additionally reduces the activity or concentration of WLD^{S} within axons or synapses in some way, then its protective capacity might be diminished. This could apply to disorders of axonal transport, protein synthesis, or protein turnover, among others.

Our findings have therapeutic implications for human disorders. Specifically, they suggest that strategies directed at blockade of SARM1 function have the potential to be more effective than WLD^{S}-related therapies in neuromuscular synaptopathies and potentially in a broader group of neurodegenerative disorders. In addition, the remarkable survival and health of *Nmnat2*$^{gtE/gtE}$;*Sarm1*$^{-/-}$ mice into old age suggests that even long-term therapeutic interventions based on blocking SARM1 function might be both effective and well tolerated by patients.

This study confirms SARM1 as a key regulator of degeneration caused by a NMNAT2 deficiency. *Sarm1* deletion appears to block this process indefinitely without affecting long-term survival, despite the predicted substantial reduction in nicotinamide adenine dinucleotide (NAD)-synthesizing capacity (Gilley et al., 2015). SARM1 has been shown to possess NADase activity that promotes injury-induced axon or synapse degeneration and can be inhibited by NMNAT activity (Gerdts et al., 2015; Sasaki et al., 2016; Essuman et al., 2017). Therefore, a model in which survival depends on NMNAT-dependent NAD production balancing NAD consumption, including any resulting from constitutive SARM1 NADase activity, is attractively simple. However, NAD consumption in uninjured *Sarm1*$^{-/-}$ axons has been shown to be comparable to wild-type consumption, suggesting that SARM1 NADase activity under normal conditions is minimal (Sasaki et al., 2016). Instead, a situation in which a loss of NMNAT activity results in the upregulation of SARM1 NADase activity via intermediate signals and/or interactions to trigger degeneration is more consistent with current findings (Sasaki et al., 2016; Di Stefano et al., 2017).

Finally, the similarity between the progressive phenotype in *Nmnat2*$^{gtE/gtE}$;*Wld*$^{S/S}$ mice and some mouse models of ALS is intriguing. While there is, as yet, no established link between NMNAT2 and ALS, the *SARM1* locus has been associated with sporadic ALS (Fogh et al., 2014), hinting at involvement of Wallerian-like mechanisms. The neuromuscular defect in *Nmnat2*$^{gtE/gtE}$;*Wld*$^{S/S}$ mice could thus model some aspects of ALS disease pathogenesis. Even if the underlying mechanisms are unrelated, *Nmnat2*$^{gtE/gtE}$;*Wld*$^{S/S}$ mice would be a useful tool for assessing reversibility of ALS-like symptoms, because silencing of the *Nmnat2*^{gtE} gene trap allele is reversible (Gilley et al., 2013).

EXPERIMENTAL PROCEDURES

Mouse Breeding and Maintenance

Animal work was performed in accordance with the 1986 Animals (Scientific Procedures) Act under Project License PPL 70/7620 following an appropriate ethical review process at the Babraham Institute. Genotyping for the *Nmnat2*^{gtE}, *Wld*^{S}, and *Sarm1* knockout alleles was performed as described previously (Gilley et al., 2013, 2015). Littermates were used where possible. The ages and genders of mice used in individual experiments are described in the figure legends.

RT-PCR

Semiquantitative endpoint RT-PCR was used to confirm *Nmnat2* gene silencing in the brains of *Nmnat2*$^{gtE/gtE}$;*Wld*$^{S/S}$ and *Nmnat2*$^{gtE/gtE}$;*Sarm1*$^{-/-}$ mice aged 10–12 months essentially as described previously (Gilley et al., 2013).

Accelerating Rotarod Task

Locomotor performance was tested on an accelerating Rotarod (Ugo Basile, Model 7650, Varese, Italy). Mice were familiarized with the apparatus (two 5 min runs at 10 rpm) one day before testing. At each test age, mice performed three 5 min trials (3 to 30 rpm) separated by 30 min rests. Latency to fall (max 300 s) was recorded. Only involuntary falls were scored. Mice dismounting voluntarily were placed back onto the apparatus once, but the run was excluded from the analysis if repeated. Best trial performance was used for statistical analyses.

H&E Staining

Transverse cryosections of gastrocnemius muscles snap frozen in liquid nitrogen-chilled isopentane (8 μm thickness) or fixed in 4% paraformaldehyde (20 μm thickness) were stained with H&E as previously described (Gilley et al., 2013). Images were captured using a MicroPublisher camera (QImaging) on an Olympus BX50 microscope (20× objective). Staining of snap-frozen muscle sections was optimal for visualization of muscle structure without the artifactual muscle fiber separation seen on fixed sections.

NMJ Innervation

Innervation of NMJs in gastrocnemius and FDB muscles was assessed by immunofluorescent staining. Staining was performed essentially as described previously (Krieger et al., 2013) on whole-mount muscles or longitudinal cryosections (60 μm thickness). Confocal z stack series were acquired using Olympus FV1000 or Leica SPE scanning laser confocal microscopes (20× or 40× objectives). Multiple z stack series were acquired for each muscle, and z projections were generated for analysis. Endplate occupancy was determined by assessing the extent of overlap or direct abuttal of βIII-tubulin staining (axon terminal) with α-bungarotoxin staining (endplate). Endplates were scored as denervated when essentially none of the endplate (less than ~5%) was deemed to be occupied by the axon terminal, fully innervated with complete (greater than ~95%) occupancy, and partially innervated with intermediate occupancy (observer determined, scored blind). Original z stack series were examined to exclude chance overlay of proximal axon segments and endplates in non-adjacent focal planes.

Isometric Muscle Tension Recordings

Force measurements for FDB muscle were made from FDB muscle-tibial nerve preparations as described previously (Beirowski et al., 2009), except that the proximal tendon was connected to a MLT0202 (0–25 g) isometric force transducer (AD Instruments, Oxford, UK) and the tibial nerve was stimulated using 0.1–0.2 ms pulses of up to 10 V using a Digitimer DS2 isolated stimulator (Digitimer, Welwyn Garden City, UK) triggered via a Powerlab 26T interface. Tension responses were digitized at 1 kHz using Chart 7 or Scope 4 software (all ADInstruments).

Counts of Myelinated Tibial Nerve Axons

Transverse sections (20 μm) of fixed calf (from mid-way between knee and ankle) were stained with FluoroMyelin red according to the manufacturer's instructions (Life Technologies). Images of tibial nerves were captured on an Olympus FV1000 point scanning confocal microscope imaging system (40× objective). Axon counts (inferred from numbers of myelin sheaths) were performed blind using the multi-point selection tool in ImageJ.

Statistical Analysis

Appropriate statistical testing of data was performed using Prism (GraphPad Software, La Jolla, USA). Tests are described in the figure legends. A p value < 0.05 was considered significant.

DATA AND SOFTWARE AVAILABILITY

Source data for graphs can be found in the the University of Cambridge Repository (Apollo) at https://doi.org/10.17863/CAM.13389.

SUPPLEMENTAL INFORMATION

Supplemental Information includes two figures, one table, and six movies and can be found with this article online at https://doi.org/10.1016/j.celrep.2017.09.027.

AUTHOR CONTRIBUTIONS

Conceptualization, J.G. and M.P.C.; Methodology, J.G. and R.R.R.; Investigation, J.G. and R.R.R.; Writing – Original Draft, J.G.; Writing – Review & Editing, J.G., R.R.R., and M.P.C.; Visualization, J.G.; Funding Acquisition, M.P.C. and R.R.R.; Supervision, M.P.C.

ACKNOWLEDGMENTS

We thank Robert Chou for technical help optimizing NMJ staining, Andrea Loreto for comments on the manuscript, Babraham Institute animal facility staff for providing movies of mice, and Dr. Anne Segonds-Pichon for help with statistical analyses. This work was funded by an Institute Strategic Programme grant from the Biotechnology and Biological Sciences Research Council, Medical Research Council grants MR/N004582/1 and MR/M024075/1, and Motor Neurone Disease Association (MNDA) grant 838-791.

Received: May 2, 2017
Revised: August 25, 2017
Accepted: September 7, 2017
Published: October 3, 2017

REFERENCES

Ali, Y.O., Allen, H.M., Yu, L., Li-Kroeger, D., Bakhshizadehmahmoudi, D., Hatcher, A., McCabe, C., Xu, J., Bjorklund, N., Taglialatela, G., et al. (2016). NMNAT2:HSP90 complex mediates proteostasis in proteinopathies. PLoS Biol. *14*, e1002472.

Beirowski, B., Babetto, E., Gilley, J., Mazzola, F., Conforti, L., Janeckova, L., Magni, G., Ribchester, R.R., and Coleman, M.P. (2009). Non-nuclear Wld(S) determines its neuroprotective efficacy for axons and synapses in vivo. J. Neurosci. *29*, 653–668.

Cohen, M.S., Ghosh, A.K., Kim, H.J., Jeon, N.L., and Jaffrey, S.R. (2012). Chemical genetic-mediated spatial regulation of protein expression in neurons reveals an axonal function for wld(s). Chem. Biol. *19*, 179–187.

Conforti, L., Gilley, J., and Coleman, M.P. (2014). Wallerian degeneration: an emerging axon death pathway linking injury and disease. Nat. Rev. Neurosci. *15*, 394–409.

Di Stefano, M., Loreto, A., Orsomando, G., Mori, V., Zamporlini, F., Hulse, R.P., Webster, J., Donaldson, L.F., Gering, M., Raffaelli, N., et al. (2017). NMN deamidase delays Wallerian degeneration and rescues axonal defects caused by NMNAT2 deficiency in vivo. Curr. Biol. *27*, 784–794.

Essuman, K., Summers, D.W., Sasaki, Y., Mao, X., DiAntonio, A., and Milbrandt, J. (2017). The SARM1 Toll/interleukin-1 receptor domain possesses intrinsic NAD+ cleavage activity that promotes pathological axonal degeneration. Neuron *93*, 1334–1343.

Fischer, L.R., Culver, D.G., Davis, A.A., Tennant, P., Wang, M., Coleman, M., Asress, S., Adalbert, R., Alexander, G.M., and Glass, J.D. (2005). The WldS gene modestly prolongs survival in the SOD1G93A fALS mouse. Neurobiol. Dis. *19*, 293–300.

Fogh, I., Ratti, A., Gellera, C., Lin, K., Tiloca, C., Moskvina, V., Corrado, L., Sorarù, G., Cereda, C., Corti, S., et al.; SLAGEN Consortium and Collaborators (2014). A genome-wide association meta-analysis identifies a novel locus at 17q11.2 associated with sporadic amyotrophic lateral sclerosis. Hum. Mol. Genet. *23*, 2220–2231.

Gerdts, J., Summers, D.W., Sasaki, Y., DiAntonio, A., and Milbrandt, J. (2013). Sarm1-mediated axon degeneration requires both SAM and TIR interactions. J. Neurosci. *33*, 13569–13580.

Gerdts, J., Brace, E.J., Sasaki, Y., DiAntonio, A., and Milbrandt, J. (2015). SARM1 activation triggers axon degeneration locally via NAD^+ destruction. Science *348*, 453–457.

Gilley, J., and Coleman, M.P. (2010). Endogenous Nmnat2 is an essential survival factor for maintenance of healthy axons. PLoS Biol. *8*, e1000300.

Gilley, J., Adalbert, R., Yu, G., and Coleman, M.P. (2013). Rescue of peripheral and CNS axon defects in mice lacking NMNAT2. J. Neurosci. *33*, 13410–13424.

Gilley, J., Orsomando, G., Nascimento-Ferreira, I., and Coleman, M.P. (2015). Absence of SARM1 rescues development and survival of NMNAT2-deficient axons. Cell Rep. *10*, 1974–1981.

Gillingwater, T.H., Thomson, D., Mack, T.G., Soffin, E.M., Mattison, R.J., Coleman, M.P., and Ribchester, R.R. (2002). Age-dependent synapse withdrawal at axotomised neuromuscular junctions in Wld(s) mutant and Ube4b/Nmnat transgenic mice. J. Physiol. *543*, 739–755.

Krieger, F., Elflein, N., Ruiz, R., Guerra, J., Serrano, A.L., Asan, E., Tabares, L., and Jablonka, S. (2013). Fast motor axon loss in SMARD1 does not correspond to morphological and functional alterations of the NMJ. Neurobiol. Dis. *54*, 169–182.

Ljungberg, M.C., Ali, Y.O., Zhu, J., Wu, C.S., Oka, K., Zhai, R.G., and Lu, H.C. (2012). CREB-activity and nmnat2 transcription are down-regulated prior to neurodegeneration, while NMNAT2 over-expression is neuroprotective, in a mouse model of human tauopathy. Hum. Mol. Genet. *21*, 251–267.

Loreto, A., Di Stefano, M., Gering, M., and Conforti, L. (2015). Wallerian degeneration is executed by an NMN-SARM1-dependent late Ca(2+) influx but only modestly influenced by mitochondria. Cell Rep. *13*, 2539–2552.

Mack, T.G., Reiner, M., Beirowski, B., Mi, W., Emanuelli, M., Wagner, D., Thomson, D., Gillingwater, T., Court, F., Conforti, L., et al. (2001). Wallerian degeneration of injured axons and synapses is delayed by a Ube4b/Nmnat chimeric gene. Nat. Neurosci. *4*, 1199–1206.

Mi, W., Beirowski, B., Gillingwater, T.H., Adalbert, R., Wagner, D., Grumme, D., Osaka, H., Conforti, L., Arnhold, S., Addicks, K., et al. (2005). The slow Wallerian degeneration gene, WldS, inhibits axonal spheroid pathology in gracile axonal dystrophy mice. Brain *128*, 405–416.

Osterloh, J.M., Yang, J., Rooney, T.M., Fox, A.N., Adalbert, R., Powell, E.H., Sheehan, A.E., Avery, M.A., Hackett, R., Logan, M.A., et al. (2012). dSarm/Sarm1 is required for activation of an injury-induced axon death pathway. Science *337*, 481–484.

Oyebode, O.R., Hartley, R., Singhota, J., Thomson, D., and Ribchester, R.R. (2012). Differential protection of neuromuscular sensory and motor axons and their endings in Wld(S) mutant mice. Neuroscience *200*, 142–158.

Sasaki, Y., Nakagawa, T., Mao, X., DiAntonio, A., and Milbrandt, J. (2016). NMNAT1 inhibits axon degeneration via blockade of SARM1-mediated NAD(+) depletion. eLife *5*, e19749.

Valdez, G., Tapia, J.C., Kang, H., Clemenson, G.D., Jr., Gage, F.H., Lichtman, J.W., and Sanes, J.R. (2010). Attenuation of age-related changes in mouse neuromuscular synapses by caloric restriction and exercise. Proc. Natl. Acad. Sci. USA *107*, 14863–14868.

Vande Velde, C., Garcia, M.L., Yin, X., Trapp, B.D., and Cleveland, D.W. (2004). The neuroprotective factor Wlds does not attenuate mutant SOD1-mediated motor neuron disease. Neuromolecular Med. *5*, 193–203.

Walker, L.J., Summers, D.W., Sasaki, Y., Brace, E.J., Milbrandt, J., and DiAntonio, A. (2017). MAPK signaling promotes axonal degeneration by speeding the turnover of the axonal maintenance factor NMNAT2. eLife *6*, e22540.

Zhu, X., Libby, R.T., de Vries, W.N., Smith, R.S., Wright, D.L., Bronson, R.T., Seburn, K.L., and John, S.W. (2012). Mutations in a P-type ATPase gene cause axonal degeneration. PLoS Genet. *8*, e1002853.

Cell Reports, Volume *21*

Supplemental Information

Sarm1 Deletion, but Not *Wld*^{S}, Confers Lifelong Rescue in a Mouse Model of Severe Axonopathy

Jonathan Gilley, Richard R. Ribchester, and Michael P. Coleman

cross	viable offspring (>P10)		
	Genotype	***Obs.***	*Exp.*
Nmnat2$^{+/gtE}$;Wld$^{S/S}$ x *Nmnat2$^{+/gtE}$;Wld$^{S/S}$*	*Wld$^{S/S}$*	**67**	68.5
	Nmnat2$^{+/gtE}$;Wld$^{S/S}$	**144**	137
	Nmnat2$^{gtE/gtE}$;Wld$^{S/S}$	**63**	68.5

χ^2 test $p = 0.660$

cross	viable offspring (>P10)		
	Genotype	***Obs.***	*Exp.*
Nmnat2$^{+/gtE}$;Sarm1$^{-/-}$ x *Nmnat2$^{+/gtE}$;Sarm1$^{-/-}$*	*Sarm1$^{-/-}$*	**32**	31.25
	Nmnat2$^{+/gtE}$;Sarm1$^{-/-}$	**61**	62.5
	Nmnat2$^{gtE/gtE}$;Sarm1$^{-/-}$	**32**	31.25

χ^2 test $p = 0.965$

Table S1. Mendelian ratios of viable offspring from *Nmnat2$^{+/gtE}$;Wld$^{S/S}$* and *Nmnat2$^{+/gtE}$;Sarm1$^{-/-}$* crosses (updated). Related to Figure 1.

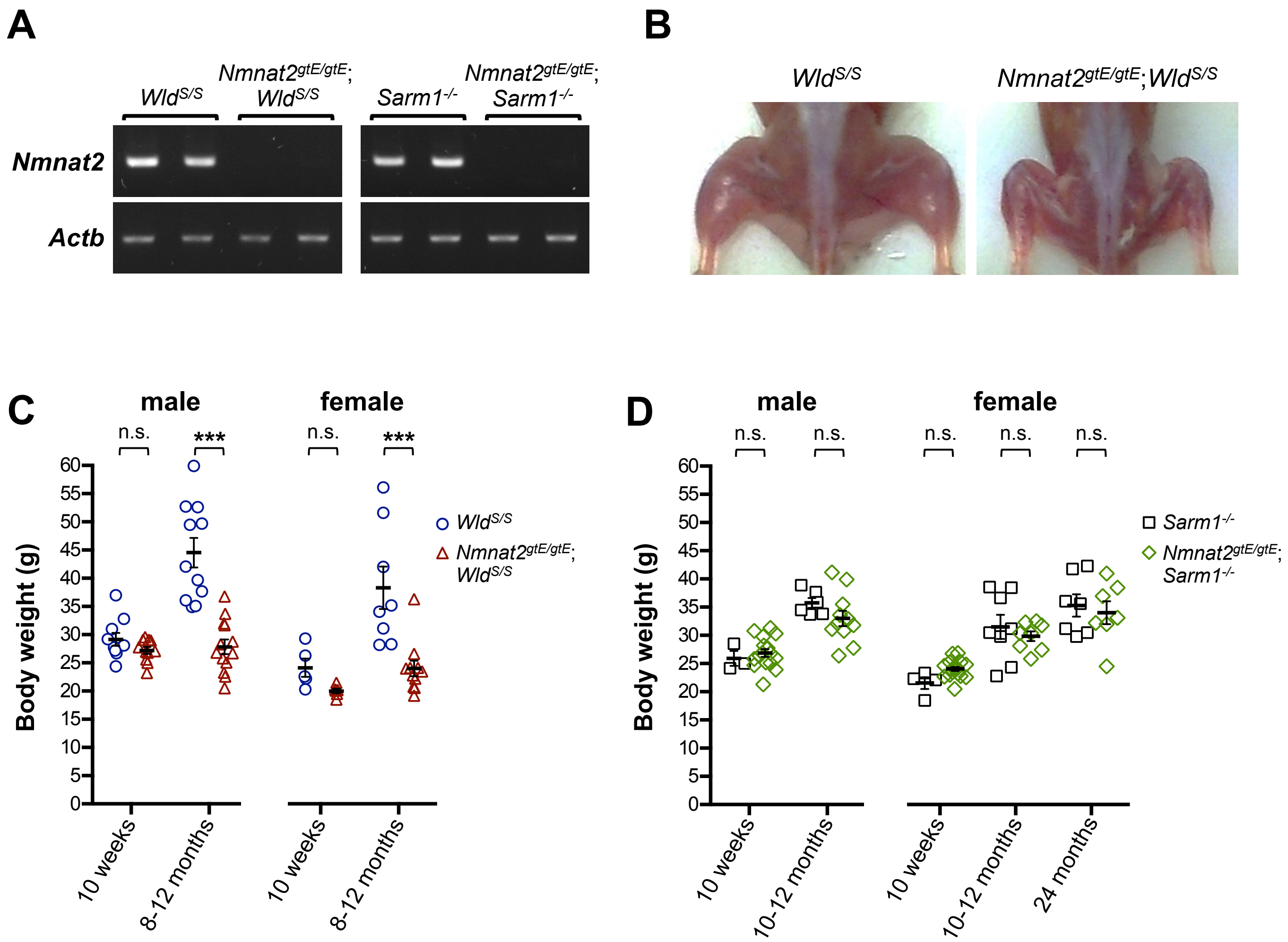

Figure S1. Muscle atrophy and an absence of weight gain in *Nmnat2*$^{gtE/gtE}$;*Wld*$^{S/S}$ mice but not *Nmnat2*$^{gtE/gtE}$;*Sarm1*$^{-/-}$ mice. Related to Figure 1. (**A**) RT-PCR analysis of *Nmnat2* mRNA levels in brains of 10 to 12-month-old mice of the indicated genotypes. *Actb* acts as a sample reference. Images are representative of n = 3 mice per genotype. Cycle numbers were adjusted for *Nmnat2* detection such that band intensity is close to saturation for the *Wld*$^{S/S}$ and *Sarm1*$^{-/-}$ control samples to emphasize the relative absence of *Nmnat2* mRNA in the *Nmnat2*$^{gtE/gtE}$;*Wld*$^{S/S}$ and *Nmnat2*$^{gtE/gtE}$;*Sarm1*$^{-/-}$ samples. (**B**) Widespread hindlimb muscle wasting in *Nmnat2*$^{gtE/gtE}$;*Wld*$^{S/S}$ mice relative to matched *Wld*$^{S/S}$ controls. Representative 6-month-old female mice are shown but the phenotype invariantly develops from 3-5 months onwards in both male and female *Nmnat2*$^{gtE/gtE}$;*Wld*$^{S/S}$ mice. (**C** and **D**) Body weights of male and female *Wld*$^{S/S}$ and *Nmnat2*$^{gtE/gtE}$;*Wld*$^{S/S}$ mice (**C**) and *Sarm1*$^{-/-}$ and *Nmnat2*$^{gtE/gtE}$;*Sarm1*$^{-/-}$ mice (**D**) at the indicated ages. Individual values with means ±SEM are plotted (n.s. not significant [$p > 0.05$] or ***$p < 0.001$ in two-way ANOVA with Bonferroni's multiple comparisons, separate tests for males and females).

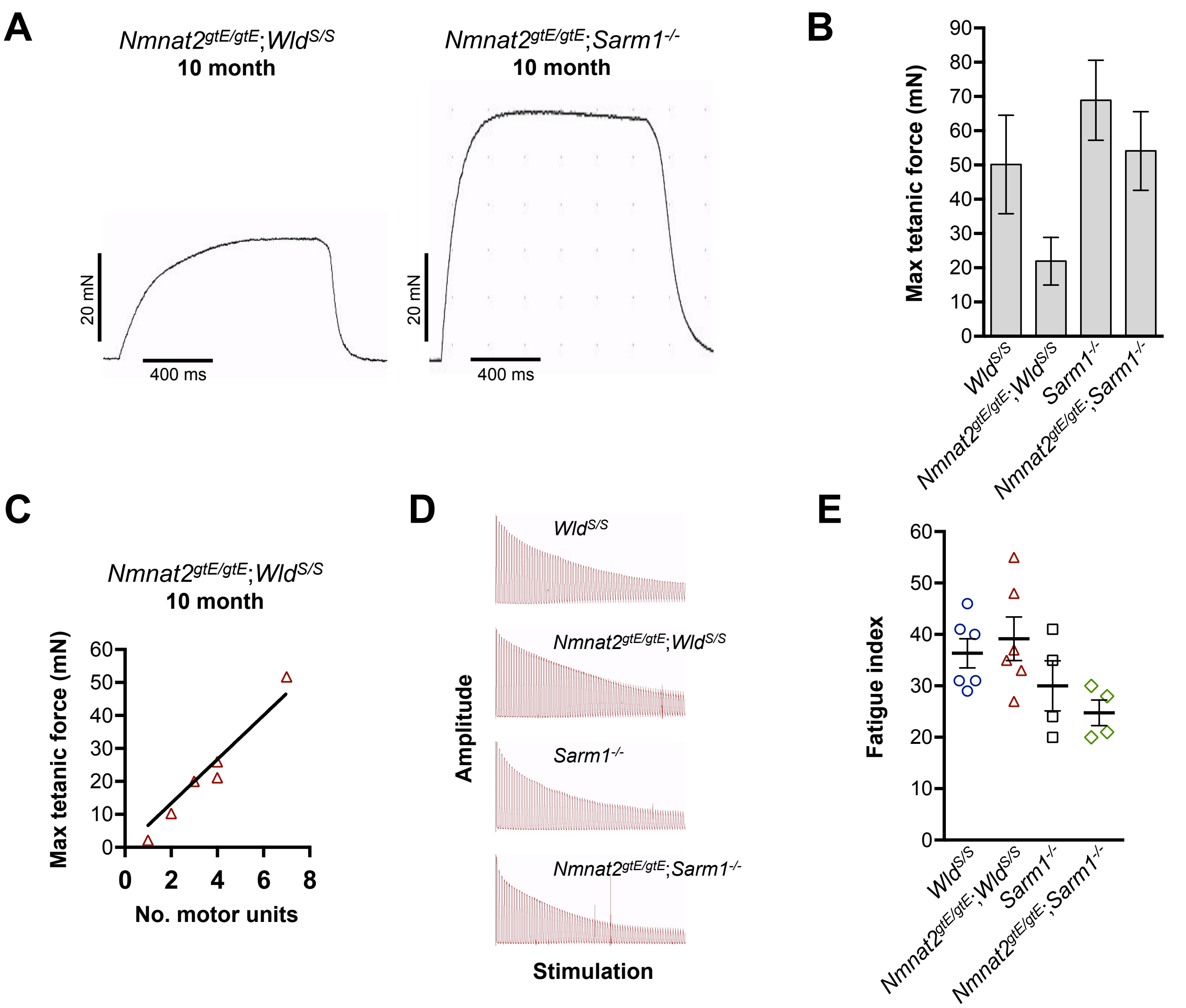

Figure S2. Motor unit numbers are reduced in 10 month *Nmnat2$^{gtE/gtE}$;Wld$^{S/S}$* FDB muscles but remaining units function normally. Related to Figure 2. (**A**) Representative maximum tetanic tension response (50 Hz stimulation for 1 s) of FDB muscle preparations from 10-month-old male *Nmnat2$^{gtE/gtE}$;Wld$^{S/S}$* and *Nmnat2$^{gtE/gtE}$;Sarm1$^{-/-}$* mice. (**B**) Maximum tetanic force of FDB muscles from10-month-old male mice of the genotypes listed. Data are mean ±SEM (n = 6 muscles from three mice, except for n = 4 muscles from two *Sarm1$^{-/-}$* mice). *Nmnat2$^{gtE/gtE}$;Wld$^{S/S}$* force is noticeably reduced compared to the other genotypes due to reduced numbers of motor units (Fig. 3E), although substantial intra-genotype variation means that group sizes are not sufficient for statistical significance (one-way ANOVA, $p > 0.05$). (**C**) Correlation between (reduced) numbers of motor units in *Nmnat2$^{gtE/gtE}$;Wld$^{S/S}$* FDB muscle explants and maximum tetanic force. Spearman correlation $r = 0.986$ ($p = 0.0056$). This suggests that, although different numbers of motor units remain in individual 10-month-old *Nmnat2$^{gtE/gtE}$;Wld$^{S/S}$* FDB muscles, those that are present are similar in size. (**D**) Representative fatigue profiles of FDB muscle explants of the four genotypes studied (as indicated) elicited by 50 Hz tetanic stimulation for 3 s, at intervals of 5s (gaps between each tetanus were clipped). (**E**) Fatigue resistance of FDB muscle explants for 10-month-old mice of the genotypes listed. Individual values with means ±SEM are plotted (same groups sizes as in B). The index of fatigue resistance is the stimulus number that produced a tetanic force equal to 50% of the initial force. Fatigueability of *Nmnat2$^{gtE/gtE}$;Wld$^{S/S}$* and *Nmnat2$^{gtE/gtE}$;Sarm1$^{-/-}$* FDB muscles is not significantly different from matched *Wld$^{S/S}$* or *Sarm1$^{-/-}$* controls (one-way ANOVA, $p > 0.05$). However, grouping muscles based on *Wld$^{S/S}$* or *Sarm1$^{-/-}$* status (i.e. excluding *Nmnat2* status) suggests genetic background does influence fatigueability (*t* test for *Wld$^{S/S}$* and *Nmnat2$^{gtE/gtE}$;Wld$^{S/S}$* versus *Sarm1$^{-/-}$* and *Nmnat2$^{gtE/gtE}$;Sarm1$^{-/-}$*, $p = 0.0126$).
